# Hip Arthroscopy for Femoroacetabular Impingement Syndrome Improves Patient‐Reported Outcome Scores Regardless of Smoking Status

**DOI:** 10.1002/ars2.70035

**Published:** 2026-05-18

**Authors:** Omkar N. Prabhavalkar, Hashim J. F. Shaikh, Patrick Castle, Sarah Wegman, Richard Lander, Justin Harrington, Urvi J. Patel, Brian D. Giordano

**Affiliations:** ^1^ Department of Orthopaedic Surgery & Physical Performance University of Rochester Rochester New York U.S.A.; ^2^ Department of Orthopaedics, Division of Sports Medicine University of Rochester Rochester New York U.S.A.

## Abstract

**Purpose:**

To compare outcomes of hip arthroscopy using Patient‐Reported Outcomes Measurement Information System (PROMIS) scores in patients with a history of smoking versus nonsmokers.

**Methods:**

Patients who underwent hip arthroscopy at our institution between January 2015 and June 2024 and had PROMIS data including physical function (PF), pain interference (PI), and depression scores were collected and split into 2 groups based on smoking history. Patients were included in this study if they were aged ≥18 years at the time of surgery and underwent hip arthroscopy for the treatment of femoroacetabular impingement syndrome, with preoperative PROMIS and postoperative PROMIS scores ≥1 year. The group of smokers was then propensity‐matched to the group of nonsmokers based on age at surgery, body mass index, sex, race, and insurance status. Continuous variables were calculated as mean ± standard deviation and compared using 2‐tailed *t*‐tests while categorical variables were compared using chi‐square analysis between the 2 groups. Mean clinically important difference was defined as one‐half the standard deviation of preoperative PROMIS scores. Patient acceptable symptomatic state thresholds were 51.8 for PF and 51.9 for PI.

**Results:**

Two hundred eighty‐seven nonsmokers and 88 smokers met inclusion criteria and had minimum 1‐year follow‐up. Eighty‐seven smokers (87 hips) were propensity‐matched to 171 nonsmokers (171 hips). The mean follow‐up time was 28.4 ± 16.2 months. There were no differences in the groups with regard to sex (*P* = .88), age at surgery (*P* = .25), race (*P* = .50), body mass index (*P* = .88), or insurance status (*P* = .96). There were no significant differences in procedures such as labral repairs, femoroplasties, or acetabuloplasties performed. Both groups experienced improvement from preoperative to postoperative PROMIS scores for all domains. The nonsmokers group showed significantly greater preoperative PF (*P* = .0053), PI (*P* = .0024), and depression (*P* < .001) scores and significantly greater postoperative PF (*P* = .0043), PI (*P* = .017), and depression (*P* = .0019) scores. No differences were seen in the achievement of mean clinically important difference between groups (PF: 52.9% vs 52.6%, *P* = .97; PI: 31% vs 33.9%, *P* = .64). No differences were seen in patient acceptable symptomatic state between groups (PF: *P* = .23, PI: *P* = .77).

**Conclusions:**

Patients with a smoking history showed significant improvement in PROMIS scores following hip arthroscopy. Smokers began and ended recovery with lower PROMIS scores, indicating that smoking affects baseline status and final outcomes, despite comparable improvement. No differences were seen in the achievement of mean clinically important difference.

**Level of Evidence:**

Level III, retrospective comparative study.

Hip arthroscopy has emerged as a widely used, minimally invasive surgical intervention for addressing intra‐articular hip pathology, particularly femoroacetabular impingement syndrome (FAIS). As surgical techniques and diagnostic capabilities have evolved, the prevalence of hip arthroscopy has significantly increased over the past 2 decades.^1^ Numerous studies have shown improvements in postoperative outcomes across diverse patient populations, reinforcing its role in managing hip pain and restoring function.^2^, ^3^, ^4^ With its growing use, there has been increasing interest in identifying patient‐specific demographic and clinical factors that may influence surgical outcomes.

Among these factors, smoking status has long been recognized as a negative prognostic indicator across a range of medical and surgical conditions. A history of smoking is associated with impaired wound healing, increased infection risk, delayed bone union, and overall poorer surgical outcomes.^5^, ^6^, ^7^ These effects are particularly well documented in orthopaedic surgery, where smoking has been linked to complications in procedures such as spinal fusion, joint replacement, and fracture repair.^8^, ^9^, ^10^

To more accurately evaluate patient‐reported outcomes after orthopaedic procedures, the use of the Patient‐Reported Outcomes Measurement Information System (PROMIS) has gained popularity. PROMIS scores, which include measures such as physical function (PF), pain interference (PI), and depression (Dep.), offer a validated, standardized approach to capturing meaningful changes in patient health and quality of life. Compared with legacy scoring systems such as Nonarthritic Hip Score, modified Harris Hip Score, Hip Outcome Score, and International Hip Outcome Tool, PROMIS is more sensitive to changes over time and is well suited for analyzing outcomes in large patient cohorts.^11^, ^12^

The purpose of this study was to compare outcomes of hip arthroscopy using PROMIS scores in patients with a history of smoking versus nonsmokers. We hypothesized that patients with a history of smoking will report worse PROMIS scores compared with nonsmokers.

## METHODS

### Cohort Summary

The University Rochester Medical Center Institutional Review Board (IRB) approved this retrospective study with a waiver of informed consent (STUDY00008086). Patients who underwent hip arthroscopy between the dates of January 2015 and June 2024 were identified by searching the Current Procedural Terminology codes 29861, 29862, 29863, 29914, 29915, 29916, and 29999. For patients treated for FAIS specifically, at least 1 of the FAIS‐related codes (29914, 29915, or 29916) was required to confirm inclusion. The additional Current Procedural Terminology codes were included to ensure a comprehensive search and to capture cases that might have been coded with secondary or adjunct arthroscopic procedures. Patients were included in this study if they were aged 18 years or older at the time of surgery and underwent hip arthroscopy for the treatment of FAIS with or without labral tear. All patients had an alpha angle of >55. Exclusion criteria included dysplastic patients, Workers’ Compensation patients, failure to complete a PROMIS questionnaire prior to surgery, or failure to complete a PROMIS questionnaire at or after 1 year after surgery.

### PROMIS Outcomes

Patients completed PROMIS questionnaires on an electronic tablet (Apple, Cupertino, USA) during their initial clinic visit. The domains assessed included PF, PI, and Dep. Preoperative PROMIS scores were defined as the scores obtained closest to the surgery date, within a 6‐month window. Postoperative scores were recorded at each follow‐up visit. PROMIS scores are standardized, with a mean of 50 and standard deviation of 10.^13^ Additionally, scores were categorized into 3 symptom severity levels: within normal limits, mild to moderate, and severe.^14^

### Medical Record Review and Smoking Status

Retrospective medical record review was conducted to collect demographic variables for patients including age at surgery, sex, body mass index, race, and smoking status. Procedures that were performed during surgery such as labral repair, femorplasty, and acetabuloplasty were recorded. The type of labral tear that was observed during diagnostic arthroscopy was also recorded. The Seldes classification system was used to describe labral tears, with type 1 referring to detachment of the labrum off the cartilage surface and type 2 referring to tearing or fraying within the labrum.^15^ Patients were split into 2 groups according to their current and prior smoking status. Patients who were current smokers or had any history of smoking were placed in the smokers group, and patients with no history of smoking were placed in the nonsmokers group.

### Statistical Analysis

Statistical analysis was performed using RStudio Version 1.2.5033. The 2 groups were propensity‐matched to each other on the basis of age at surgery, body mass index, race, gender, and insurance status in a 1:2 ratio. A nearest neighbor approach was used with a 0.1 caliper. An a priori power analysis indicated that 43 patients in the smaller cohort and 86 in the larger cohort were required to achieve 80% power with a significance level of 0.05, assuming a PROMIS mean clinically important difference (MCID) improvement of 4.0 points.^16^ MCID was defined as ½ of the standard deviation of the preoperative scores. Patient acceptable symptomatic state (PASS) analysis was also conducted, with PASS threshold set at 51.8 for PF and 51.9 for PI.^17^ For all demographic information and patient‐reported outcomes, these data were reported as mean ± standard deviation. To compare the preoperative and postoperative scores within each group, the Wilcoxon‐signed rank test was used, and 2‐tailed *t*‐tests were used to compare means between the groups. All categorical variables were compared using chi‐square analysis. Statistical significance was set at a value of *P* < .05.

## RESULTS

### Patient Characteristics

A total of 375 patients, 88 smokers and 287 nonsmokers, met inclusion criteria and had minimum 1‐year follow‐up. The mean follow‐up time was 28.4 ± 16.2 months. Out of these patients, 87 smokers (87 hips) were propensity‐matched to 171 nonsmokers (171 hips). Table 1 presents a full summary of patient demographics for each group. There were no significant differences seen with regard to sex (*P* = .88), age at surgery (*P* = .25), race (*P* = .50), body mass index (*P* = .88), or insurance status (*P* = .96) (Table 1).

**TABLE 1:** Patient Demographics

	Smokers(n = 87)	Nonsmokers (n = 171)	*P* Value
**Sex**			.88
Female	50	100	
Male	37	71	
**Age at surgery**	37.9 ± 10.2	36.4 ± 11.5	.25
**Race**			.50
White or Caucasian	80	161	
Black/African American	0	0	
Other	7	10	
**BMI**	27.5 ± 5.4	27.3 ± 6.9	.88
**Insurance**			.96
Commercial	71	142	
Medicare/Medicaid	15	27	
Workers’ Compensation	1	2	

BMI, body mass index.

### Intraoperative Findings and Procedures

There were no differences seen in the types of labral tears observed (*P* = .31), labral repairs performed (*P* = .43), acetabuloplasties performed (*P* = .79), or femoroplasties performed (*P* = .80). Table 2 shows the intraoperative findings and procedures performed between groups.

**TABLE 2:** Procedures and Intraoperative Findings

	Smokers (n = 87)	Nonsmokers (n = 171)	*P* Value
Labral repair	68 (78.2%)	126 (73.6%)	.43
Femoroplasty	84 (96.6%)	164 (95.9%)	.80
Acetabuloplasty	69 (79.3%)	138 (80.7%)	.79
**Seldes type**			.31
Type 1	66 (75.9%)	123 (71.9%)	
Type II	5 (5.7%)	20 (11.7%)	
Degenerative	16 (18.4%)	28 (16.3%)	

### Patient‐Reported Outcomes, MCID, and PASS Analysis

With regard to PROMIS outcome scores, both groups showed improvement from preoperative to postoperative scores (smokers: PF—*P* < .001, PI—*P* = .015, Dep.—*P* < .001; nonsmokers: PF—*P* < .001, PI—*P* = .0099, Dep.—*P* < .001). Between groups, the nonsmokers had significantly better preoperative scores for PF (*P* = .00529), PI (0.00243), and Dep. (*P* < .001) (Table 3). The nonsmokers also showed significantly better postoperative scores for PF (*P* = .00431), PI (*P* = .0169), and Dep. (*P* = .00191). When comparing improvement, however, there were no significant differences between the groups for any of the PROMIS outcomes evaluated. Additionally, the 2 groups had similar rates of achievement of MCID for both PF (52.9% vs 52.6%, *P* = .97) and PI (31% vs 33.9%, *P* = .64) (Table 4). There were also no differences seen in the achievement of PASS for both PF (6.9% vs 11.7%, *P* = .23) and PI (16.1% vs 17.5%, *P* = .77) (Table 5).

**TABLE 3:** PROMIS Outcomes by Group

	Smokers (n = 87)	Nonsmokers (n = 171)	*P* Value
**PF**			
Preoperative	38.6 ± 6.0	40.7 ± 5.9	**.00529**
Latest	42.1 ± 8.4	44.5 ± 7.3	**.00431**
*P* value	**<.001**	**<.001**	
Improvement	3.6 ± 7.9	3.7 ± 6.7	.99
**PI**			
Preoperative	60.8 ± 10.0	57.3 ± 9.0	**.00243**
Latest	58.4 ± 8.4	56.0 ± 6.8	**.0169**
* P* value	**.015**	**.0099**	
Improvement	2.42 ± 9.1	1.28 ± 8.7	.821
**Depression**			
Preoperative	52.6 ± 11.2	47.0 ± 9.3	**<.001**
Latest	48.7 ± 12.0	43.5 ± 8.6	**.00191**
* P* value	**<.001**	**<.001**	
Improvement	3.94 ± 9.7	3.49 ± 7.4	.897

*Note*: Bold indicates statistical significance set as *P* < .05.

PF, physical function; PI, pain interference; PROMIS, Patient‐Reported Outcomes Measurement Information System.

**TABLE 4:** Comparison of PF and PI MCID Achievement Rates

	MCID Achieved	MCID *P* Value
PF (MCID threshold: 3.01)		.97
Smokers (n = 87)	46 (52.9)	
Nonsmokers (n = 171)	90 (52.6)	
PI (MCID threshold: 4.75)		.64
Smokers (n = 87)	27 (31.0)	
Nonsmokers (n = 171)	58 (33.9)	

*Note*: Values are presented as n (%).

MCID, minimal clinically important difference; PF, physical function; PI, pain interference.

**TABLE 5:** Comparison of PF and PI PASS Achievement Rates

	PASS Achieved	PASS *P* Value
PF (PASS threshold: 51.8)		.23
Smokers (n = 87)	6 (6.9%)	
Nonsmokers (n = 171)	20 (11.7%)	
PI (PASS threshold: 51.9)		.77
Smokers (n = 87)	14 (16.1%)	
Nonsmokers (n = 171)	30 (17.5%)	

*Note*: Values are presented as n (%).

PASS, patient acceptable symptomatic state; PF, physical function; PI, pain interference.

## DISCUSSION

The most important finding in this study is that both smokers and nonsmokers experience improvement in outcomes measures from preoperatively to postoperatively. Smokers started with lower postoperative scores and ended with worse postoperative PROMIS scores. There were no differences in MCID or PASS achievement between groups. This suggests that smoking may play a role in preoperative planning as well as postoperative recovery and expectations.

Other studies have been conducted looking at outcomes of hip arthroscopy in smokers with hip‐specific outcome measures. Lee et al. examined outcomes of hip arthroscopy in nonsmokers versus patients who had stopped smoking at least 1 month prior to surgery and found that both smokers and nonsmokers achieved significant improvement in modified Harris Hip Score, Nonarthritic Hip Score, and visual analog scale for pain.^18^ Additionally, smokers and nonsmokers achieved similar rates of MCID for the outcome scores as well. The present study is in concordance with this previous study and shows that hip arthroscopy can have beneficial outcomes in both smokers and nonsmokers.

With this in mind, another finding of this study is that nonsmokers experienced superior preoperative and postoperative outcome scores, although improvement rates were similar between groups. The significant difference in preoperative scores suggests that smokers may be starting off at a disadvantage prior to surgery, due to general adverse outcomes of smoking affecting physical function, pain, and mood.^19^, ^20^ The significant difference seen in postoperative outcomes also aligns with the findings of other studies. In a systematic review by Emara et al., nonsmokers experienced significantly better postoperative outcomes for mHHS, visual analog scale for pain, and Hip Outcome Score‐Sports Specific outcome scales.^21^ This study also showed that patients in the smoking group had higher rates of thromboembolic and infectious complications. Jimenez et al. also showed that smokers had lower functional outcomes and higher rates of secondary surgery when compared with nonsmokers at 2‐year follow‐up.^22^ These findings highlight that smoking not only places patients at a disadvantage before surgery but also is associated with poorer postoperative outcomes and higher complication rates.

These results can help inform how we counsel patients prior to hip arthroscopy surgery. Although smokers can still benefit from surgery, the preoperative and postoperative outcome scores suggest that expectations after surgery need to be appropriately set. Furthermore, studies have also shown that smoking is positively associated with the development of arthritis in the US population.^23^ These findings are important to take into consideration as development of arthritis in the hip could limit the benefits provided by an arthroscopic procedure and increase the likelihood of needing an arthroplasty in the future.^24^

This study is strengthened by propensity‐matched cohorts, standardized institutional PROMIS collection, and inclusion of multiple validated outcome domains, allowing for a comprehensive assessment of functional and psychosocial recovery after hip arthroscopy. Although other studies have been done on the impact of smoking on hip arthroscopy, the use of PROMIS scores offers important advantages because they capture a broader view of patient health. These measures reflect overall physical function as well as key psychosocial effects that can follow injury and intervention.

### Limitations

This study has limitations that should be acknowledged. First, it is a retrospective review, which carries inherent limitations including selection bias and inability to establish causality. The follow‐up time of 1 year may be short and not reflect long‐term outcomes of hip arthroscopy in smokers. Second, the investigation was conducted at a single institution, which may limit the generalizability of the results to different populations. Third, a distribution‐based approach was used to determine the MCID rather than anchor‐based method. Although this method offers a statistical threshold of change based on the specific variability of our patient population, it may not reflect the degree of improvement perceived as meaningful by patients. Anchor‐based MCID, which relies on external criteria such as patient satisfaction, may be more representative of clinically relevant information. Furthermore, preoperative lateral center‐edge angle and Tonnis grading data, as well as secondary surgery and complications information, were not available in this study. Our study did not account for vaping, a form of nicotine use that is increasingly prevalent, particularly among younger patients, or marijuana smoking.

## CONCLUSIONS

Patients with a smoking history showed significant improvement in PROMIS scores following hip arthroscopy. Smokers began and ended recovery with lower PROMIS scores, indicating that smoking affects baseline status and final outcomes, despite comparable improvement. No differences were seen in the achievement of MCID or PASS.

## DISCLOSURES

The author (B.D.G.) declares the following financial interests/personal relationships, which may be considered as potential competing interests: B.D.G. reports a relationship with Arthrex that includes consulting or advisory; Arthrex royalties and research support. The other authors (O.N.P., H.J.F.S., P.C., S.W., R.L., J.H., U.J.P.) declare that they have no known competing financial interests or personal relationships that could have appeared to influence the work reported in this paper.
